# ‘There was just no-one there to acknowledge that it happened to me as well’: A qualitative study of male partner’s experience of miscarriage

**DOI:** 10.1371/journal.pone.0217395

**Published:** 2019-05-28

**Authors:** Ellena J. Miller, Meredith J. Temple-Smith, Jade E. Bilardi

**Affiliations:** 1 Department of General Practice, University of Melbourne, Carlton, Victoria, Australia; 2 Melbourne Sexual Health Centre, Alfred Health, Carlton, Victoria, Australia; 3 Central Clinical School, Monash University, Melbourne, Victoria, Australia; University of Cape Town, SOUTH AFRICA

## Abstract

Miscarriage occurs in up to one in four pregnancies and can be a devastating event affecting both men and women. Unfortunately, the male partner’s experience of miscarriage is seldom researched, particularly within Australia. This qualitative study involved semi-structured telephone interviews with 10 Australian men, whose partners miscarried between three months and ten years ago. Participants were recruited through professional networks and support organisations. Interviews explored men’s general miscarriage experience and the support received or lacking from both healthcare providers and social networks. Online health seeking behaviour and opinions on online support were also discussed. Data was transcribed verbatim and analysed thematically. Most men described feeling significant grief following miscarriage and felt that there was little acknowledgment of their loss, both from healthcare providers and within their social networks. Feelings of sadness, devastation, powerlessness, fear, shock and a loss of identity were common. All men felt their primary role at the time of miscarriage was to support their partner. Most men did not want to burden their partner with their emotions or grief, and struggled to find people within their social networks to talk to about their loss, leading to feelings of isolation. Overall participants felt there was inadequate support offered to men affected by miscarriage. Men wanted information, informed professionals to talk to and male-orientated support networks. A website was one mechanism suggested by men which could adequately contribute to information and support needs during this time. Men are often greatly affected by miscarriage and yet there is all too often little acknowledgement or support available to them at this time. Men affected by miscarriage want and need further support, including reputable, Australian based information and resources tailored their needs.

## Introduction

Miscarriage is a very common pregnancy complication affecting up to one in four pregnancies [1]. In Australia, miscarriage is defined as the loss of a pregnancy during the first 20 weeks gestation, with pregnancy loss after 20 weeks termed a stillbirth [2]. Definitions of miscarriage differ between countries, guidelines and publications and may include gestations up to 24 weeks [3].

In recent years, an increasing amount of research and support services have been developed for parents experiencing miscarriage; however these have primarily focused on women. In a recent UK study, 25% of women were found to likely meet the criteria for post-traumatic stress disorder (PTSD) one month after miscarriage, 32% for anxiety, and 16% for depression [4]. The number for PTSD rose to 38% at three months after the loss [4]. Women have been found to struggle with the physical loss of the unborn baby and the loss of anticipated future of the child and their own identity as a parent [2, 4–10]. Partners are often women’s central support figures with evidence to show that positive partner and social support plays a crucial role in buffering women’s grief and distress at the time of miscarriage [5–9].

While women’s experiences of, and responses to miscarriage vary, there is substantial evidence to show that women commonly experience significant psychosocial morbidity following miscarriage and grieve in a similar intensity to other types of loss [2]. Feelings of anger, distress, grief, guilt and shock are very common [10–12], with studies showing it is common for pathological grief and clinical levels of anxiety and depression to develop in the weeks, months or even years following miscarriage [13–16].

Unfortunately, there has been very limited research undertaken to understand male partner’s experience of miscarriage. Available data indicates men and women have different response patterns after miscarriage. Although men generally report the same feelings as women: grief, depression, anxiety and stress, they tend to experience these less intensely and for a shorter period [17]. However, some quantitative studies have found men experience general grief levels as high, or higher than their female partners [10, 18].

It is not uncommon for men to view their role as that of the ‘supporter’, and deal with their loss less openly and employ different coping strategies to women. Previous research has shown men may employ distractions or active-avoidance behaviours such as elevated alcohol use to deal with the grief [1, 17]. This lack of expression of their distress and grieving may be influenced by a lack of recognition of men’s loss, social expectations of men to be strong or stoic, and an assumed responsibility that their only role is to be a support for their partner [1, 17].

The support people experience from within their social networks and from healthcare professionals can play an important role in shaping the experience of miscarriage. Unfortunately, past research has shown support in both areas is commonly lacking for both men and women. Women have repeatedly raised concerns around healthcare professionals’ lack of sensitivity, empathy or acknowledgement of their loss, lack of information provision, the use of impersonalized, medicalised terminology and lack of follow up care or referrals to support services [11, 19, 20]. While data is limited, men often feel their role and loss as a father is devalued by hospital staff, or their grief is not acknowledged in the same way as their partner, if at all [21]. Men often cite issues around feeling excluded or marginalized from hospital care and the provision of information and support, compared to their female partner [17, 22].

Previous literature has noted that men tend to seek medical help and utilise health care services, at a lower rate than women. Traditional ideas of masculinity and men’s gender identity has been linked to men’s health seeking behaviours, both in Australia and internationally and across various cultural groups [23, 24]. These masculine traits include stoicism, an expectation to be independent and tough and to not seek help for fear of appearing weak [23, 24]. Men who adopt these traditional beliefs have also been found more likely to smoke, have a greater risk for physical injury due to accidents, and to report an increased prevalence of anxiety, depression and maladaptive coping patterns [23]. It has been highlighted that Australian health services and health policy focusing on men’s health need to recognise the influence masculinity and gender roles has on men’s health values, behaviours and outcomes [24].

Unfortunately, limited research also shows men can experience little support from their social networks and a reluctance to actively share their loss or feelings with them. While some studies have reported men describing helpful support from family and friends [10, 22, 25, 26], others have reported men feeling ignored by their community (21) or that they had few people who they felt they could share their grief with [26, 27]. Some men have described feeling reluctant to discuss their experience with loved ones as they felt it was a personal issue or that there was an expectation to have moved on already [10, 25–27]. In a recent Australian study undertaken by Obst (2019), eight men were interviewed about their experiences of miscarriage and/or stillbirth and reported that men needed a wide range of emotional support, including formal support groups and counselling as well as encouraging men to share experiences with other male friends or fathers [28].

Beyond this recent Australian qualitative study, little is known about how Australian men experience miscarriage, the ways in which these men may express their grief or emotions post miscarriage, and the best ways to support men during this time. The aim of this study is to address the gap in Australian literature by exploring miscarriage from a male partner perspective and men’s needs for additional support. Further research in this area will not only contribute to an improved understanding of men’s experiences but a better understanding of where we need to focus our future research and support efforts.

## Methods

This study has been reported in accordance with the Consolidated criteria for reporting qualitative research (COREQ) guidelines [29].

### Ethics

Ethical approval was sought and granted by the Department of General Practice Human Ethics Advisory Group, the University of Melbourne (No. 17449961) on the 15^th^ November 2017. A distress protocol was in place in case a participant became distressed during the interview but was not required.

### Theoretical approach

A qualitative descriptive approach was used in this study to gain a greater understanding of Australian men’s lived experience of miscarriage. Qualitative description is a pragmatic approach which aims to produce straight, in-depth descriptions of participant’s experiences in their own language and from their own viewpoint. Qualitative descriptive research is particularly useful when researchers desire a comprehensive summary of phenomena and to understand the who, what and where of events, especially in settings where time and resources are limited [30, 31].

### Method, research team and Reflexivity

Semi structured interviews were undertaken by EM, a final year medical student with an interest in reproductive health. EM was not known to any participants prior to recruitment. Study participants were informed that the study was being undertaken to better understand male partners’ experience of miscarriage and the support they required. JB is a senior research fellow with a doctorate (PhD) in public health and specialises in social health research in the area of sexual and reproductive health. MTS is a Professor and Director of Research Training in primary health care and specialises in qualitative and mixed methods research in the field of sexual and reproductive health.

### Recruitment

Men were purposively recruited into the study. To be eligible men needed to reside in Australia and have an experience of miscarriage more than three months ago but less than 10 years ago. A miscarriage was defined as pregnancy loss under 20 week’s gestation. Men whose partners had experienced a miscarriage less than 3 months ago or more than 10 years ago were excluded from the study. These timeframes were arbitrarily utilised in a previous study we undertook with women [32] in an effort to minimize distress (i.e. too close to the event) or long term recall bias. Men were recruited through existing networks known to the researchers (friends, colleagues, family), previous miscarriage study participants and a study advertisement posted on the Facebook page of an Australian pregnancy loss support group ‘Bears of Hope’ (www.bearsofhope.org.au). Men interested in participating were asked to contact EM to discuss the study further and arrange a mutually convenient time for the interview.

### Data collection

All interviews were conducted by EM by telephone (as per ethics requirements) between February and April 2018. Participants were provided with a plain language statement and consent form prior to the interview and written consent was obtained. The interview schedule (S1 Appendix) comprised of a series of demographic questions and open-ended questions pertaining to men’s experience of miscarriage, the support they received from healthcare providers and social networks, their online information and support seeking behaviours at the time of the miscarriage and their views on supports needed for men affected by miscarriage. All men were offered a $50 gift card as reimbursement for their time in participating in the study.

### Data analysis

Interviews were digitally recorded and transcribed verbatim. Field notes were collected and detailed in each participant’s transcript to provide further contextual detail. Throughout the data collection period EM, MTS and JB met weekly to review the preliminary data and discuss the emerging themes and any new data arising from the interviews. During the data collection period, two additional questions regarding experiences of pregnancies subsequent to the miscarriage were added to the interview schedule. The research team met regularly to review the manuscripts and discuss themes emerging from the data. After ten interviews were completed the research team met again to review the findings and at this point decided that data saturation had been met, as no new themes were evident in the data.

A general inductive approach was adopted [33] whereby each transcript was read in full by EM multiple times and excerpts of text were coded in each transcript. The interview schedule was used as a starting place through which initial themes were identified, and as new themes emerged they were captured in the framework. A coding framework was developed in which coded text was examined for meaning, compared for similarities and differences, and categorised into themes and sub-themes. Throughout this process, a subset of the transcripts was read independently by JB and MTS—each from a different disciplinary background—to cross check coding and ensure consensus on thematic analysis. All authors agreed upon final interpretations of the data.

## Results

Sixteen men expressed an interest in the study. Four had experienced stillbirth and were ineligible for the study and two did not respond to further contact after they were emailed information about the study. Of the 10 men who participated in the study, nine were recruited through the Bears of Hope Facebook page and one through existing networks. Interview length was 18 to 57 minutes.

Men were aged 29 to 49 years old and all had experienced at least one miscarriage (n = 9) or ectopic pregnancy (n = 1), with two men also having experienced other perinatal loss. Men had experienced miscarriages between three months and eight years ago. The socio-demographic characteristics of all participants are shown in Table 1.

**Table 1:** Participant characteristics (N = 10).

Characteristic	N(%) or Median [range]
**Age**	38 [29–49]
**Education level of completion**	
Secondary school	4 (40%)
Trade diploma or certificate	1 (10%)
University undergraduate degree	2 (20%)
University postgraduate degree	3 (30%)
**Full time employment**	10 (100%)
**Country of Birth**	
Born in Australia	9 (90%)
Born outside Australia	1 (10%)
**Relationship status**	
Single	1 (10%)
Married and living with partner	9 (90%)
**Number of miscarriages experienced**	
1	4 (40%)
2	4 (40%)
More than 2	2 (20%)
**Living children at time of miscarriage**	
0	8 (80%)
1	1 (10%)
More than 1	1 (10%)
**Living children at time of interview**	
0	3 (30%)
1	4 (40%)
More than 1	3 (30%)
**Other perinatal loss experienced**	
Still birth	1 (10%)
Neonatal death	1 (10%)

Five key themes arose from the data: 1) How men felt inside; 2) Perceived role as support for partner; 3) Coping strategies; 4) Lack of healthcare support; and 5) Importance of sharing their experience. Men described significant psychosocial impacts from miscarriage including a loss of their sense of self and future aspirations, including their identify as a father and concerns around their ability to have children in the future. Men felt their key role was to support their partner at the time of miscarriage and into the future. They employed various coping mechanisms to deal with the loss including rationalising it as a biological event, keeping themselves busy and distracted, memorializing their child or thinking toward the future. Men described a lack of support and acknowledgment of their loss as a father. They recognised the importance of being able to share their experience with family and friends, through online support networks and professionals they sought out, yet felt their ability to gain support in this way was limited. Men strongly felt the need for additional male-orientated resources, in the form of online or telephone support services.

### How men felt inside

Most men described feeling significant grief following the miscarriage, with many describing their immediate emotional responses as sad, devastated or shocked. Men often described feeling the whole experience as being on a rollercoaster and they felt powerless or had no control over the outcome or their emotions at the time.

“I was pretty devastated, I started crying. I was pretty down for about a week… really, really sad. Really down. Really down on the world.”

- Participant 4, 44 years, 2 miscarriages

“…the feeling that it’s not in your hands…. You can really want it and you can do the right thing but nothing is guaranteed and that is quite hard to deal with.”

- Participant 9, 43 years, 4 miscarriages

Several men felt the experience was life changing, altering their perspective on pregnancy, parenting and even the course of their life and career.

“…you never ever get over it and you just learn how to live with it.”

- Participant 8, 32 years, 1 miscarriage

#### Loss of identity as father

Not only did men speak of the emotional difficulty of miscarriage, a number also spoke of struggling with a loss of identity as a father which often went unrecognized.

“It’s a loss. it’s a death …but it’s more about that expectation.”

- Participant 6, 49 years, 2 miscarriages

“There was just no-one there to …acknowledge that it happened to me as well…one day I saw myself as a dad, the other day I was not a dad anymore.”

- Participant 10, 29 years, 1 miscarriage

#### Concerns about future pregnancies

While men looked to the future in hope, they also had their concerns. Some men, especially those without children, described feeling anxious about their future and ability to have children or successful pregnancies again.

“…scared. scared of her situation, of what could happen …we would be running out. might be… another embryo and we only have so many that we can.”

- Participant 4, 44 years, 2 miscarriages

Others spoke about feeling like they were on an emotional roller coaster of hope and disappointment each month when trying to conceive and realising they were not pregnant, and that their future hopes and happiness were bound up with having a child. All men described feeling some anxiety or fear about future pregnancies, or not being able to enjoy their next pregnancy until the birth.

“But it’s still hard as we… reach the end of the month and you know the period comes and we’re…and my wife knows she’s definitely not pregnant…it [deep breath] kind of brings it all back up. I go “Well why not get back on?”… it’s potentially the one thing that’s going to make her feel better and make us feel better is having that child, you know.”

- Participant 1, 31 years, 1 miscarriage

“We didn’t allow ourselves to be happy…even a few months after I was always wanting to make sure she was okay…we were so affected by miscarriages that we could not enjoy the healthy pregnancy we had and the outcome you know… it took us quite a while to get rid of these feelings of “Let’s prepare ourselves for the next hit.””

- Participant 9, 43 years, 4 miscarriages

Some men felt they did not want to tell anyone about their next pregnancy too early, for fear of there being another miscarriage.

“I just wanted to kind of keep it like totally under wraps, like let’s not say nothing to anyone …at least until the 12 week mark. You know, like which is what they say that’s the safety period, but I realised there is no safety period.”

- Participant 8, 32 years, 1 miscarriage

Men often reported they were fine–or would be fine–once they were pregnant again or successfully had a child.

“…we were able to get pregnant… and things all come good…very different in hindsight. It’s easy for us to look now and say we had three children.”

- Participant 6, 49 years, 2 miscarriages

“So we became pregnant about 4 months after…that helped as well. Because then I had something else to focus my energy on. Also, you know looking at him going “If my last son had survived I wouldn’t have you” …I can’t dwell on the fact that I’ve lost.”

- Participant 10, 29 years, 1 miscarriage

### Perceived role as support for partner

Most men felt it was their role to be the ‘supporter’ for their partner and as a man they had to be ‘strong and stoic’, putting their emotional needs aside. Although they acknowledged they were not always sure of how to do this.

“My role was just to be extremely focused… I had to switch off there’s no baby any more. I’ve got to look after my wife… It was more an advocacy role …and just trying to get help.”

- Participant 3, 41 years, 3 miscarriages

A number of men spoke of having to care for their partner who became depressed or struggled with exacerbations of other mental health issues following the miscarriage.

“…my wife got diagnosed with depression…the counsellor was really good at getting me to understand what the triggers were and what situations to avoid…to help look out for her.”

- Participant 3, 41 years, 3 miscarriages

While men wanted to support their partners, it was not uncommon for men to speak of not knowing how best to do this.

“All the while you are just standing there really not knowing… having any idea of what to do, or how you can help or anything like that. Just…kind of a passenger.”

- Participant 3, 41 years, 3 miscarriages

“I didn’t know what I was doing, I sort of made it up on the spot.”

- Participant 5, 39 years, 2 miscarriages

In taking on a supporter role, men were often left not knowing what to feel, how to deal with their own grief or appropriately express their feelings, leaving them feeling alone and isolated in their grief. They worried that expressing their emotions and grief would only further burden their partner.

“I didn’t think that she was strong enough to look after herself with what I was… likely to dump on her.”

- Participant 2, 37 years, 1 miscarriage

“At the time I didn’t feel comfortable, even bringing up anything I was thinking just for that worry that she was already under enough strain as it was.”

- Participant 5, 39 years, 2 miscarriages

Some stated that they felt their grief was unacknowledged by their partners.

“…I also found that she probably didn’t know how to be there for me.”

- Participant 10, 29 years, 1 miscarriage

“I would never know how she would have felt because I can’t get pregnant, but I think I’m going through possibly an equal part. Umm I think today we don’t fully agree on that.”

- Participant 9, 43 years, 4 miscarriages

### Coping strategies

Men described varying coping mechanisms: biological explanation, distancing and distraction, memorials, and focusing on the future.

#### Biological explanation

In an effort to better understand why they might have experienced a miscarriage, men often looked for information or a rationale for why it may have occurred.

“It was a really feeling of what the hell kind of thing. Where is this coming from and …what have we done wrong? Have we drunk too much alcohol?”

- Participant 9, 43 years, 4 miscarriages

“I was able to just say in my mind, this happened because the baby wasn’t going to be in a good position or a good place so, this is a good thing… I was able to make that conclusion in my head to help me cope with it.”

- Participant 5, 39 years, 2 miscarriages

#### Distancing, distraction and suppressing grief

Other coping strategies men often found helpful included having space—often with their partner—to come to terms with the pregnancy loss in private. Keeping busy and distracted with family and work was helpful.

“…we had a bit of distance and a bit of space…to understand and come to terms with what had gone on.”

- Participant 6, 49 years, 2 miscarriages

Some men spoke of their struggle to voice their emotions or of feeling a need to suppress and internalise their grief. They often put this down to ‘a male’ way of coping, and identified it as a key difference in the way they coped with their grief compared to their female partner.

“As a male we’re probably …just like …I’ll be fine. I’ll brush myself off and I’ll be alright …but deep down you’re not.”

- Participant 8, 32 years, 1 miscarriage

#### Memorial

Several men also spoke of the importance of remembering their child through mementos and anniversaries which played an important role in coping with their loss.

“We remember on the day. We remember around the time.”

- Participant 9, 43 years, 4 miscarriages

“New South Wales government were issuing acknowledgments of life I think they’re called. Where you go to the Births, Deaths and Marriages and actually get a certificate to say that you’ve had this happen. So we got one of those for M [son] which you know …it’s little …just helps.”

- Participant 3, 41 years, 3 miscarriages

#### Focusing on the future

Many men also spoke of thinking of the future in an effort to move forward, focusing on other ways they could have children such as adoption or IVF. Most men described believing things would be better once they were a father or had another child.

“My way of getting through it was basically to focus on the future and the future prospects and you know it’s…you know, it’s fucked but you know we’ve just got to kind of move on and hey keep trying and get back on the horse so to speak.”

- Participant 1, 31 years, 1 miscarriage

### Lack of healthcare support

Most men stated that both they and their partners had experienced poor support in hospital, particularly during admission in the emergency department, which involved an overly clinical approach to the miscarriage, a lack of acknowledgement of their loss, a lack of follow up, long wait times and that their concerns regarding the health of the foetus and their pregnant partner were not taken seriously by health care staff.

Most men reported there was little to no acknowledgement of their loss as a parent in their interactions with healthcare professionals around the time of the miscarriage.

“The hospital…they always treat the mum …“she’s gone through it…that’s all we care about, you’re just the dad.””

- Participant 8, 32 years, 1 miscarriage

“Everything was given to J, I was just the body in the room.”

- Participant 2, 37 years, 1 miscarriage

Men often felt they were given poor explanations by doctors or too little information about possible causes and complications of miscarriage, the miscarriage management options and what these involved. Several men felt health care professionals assumed both they and their partner had an understanding of medical or pregnancy terminology or procedures and did not check their understanding.

“The whole process was really clinical… there wasn’t much support in terms of… understanding, trying to explain what the issues were or something like that. It was just… “yep that’s it, there’s no heartbeat. Off you go to the next room””

- Participant 3, 41 years, 3 miscarriages

Several men felt that most clinicians regarded the miscarriage as a common pregnancy related complication, and did not adequately acknowledge the loss of a child.

“…even though they were still in the womb and it wasn’t very long, but the acknowledgement that they were alive and that they mattered was extremely important to me.”

- Participant 10, 29 years, 1 miscarriage

Several men also detailed doctors trying to hide bad news or not appearing transparent and honest.

“They don’t want to tell you something that’s going to distress you in a way…they give you lots of answers that aren’t…really straight out. I’d rather just hear it straight…at least you know then. You can prepare yourself.”

- Participant 8, 32 years, 1 miscarriage

Overall, men generally reported that there was no hospital follow up, counselling referrals or pathways and poor psychological support given to men.

“There was no direction for me to go into, there was no “oh maybe you should try this, maybe you should try that” there was nothing like that. It was all directed at her.”

- Participant 7, 32 years, 1 miscarriage

This included missed opportunities for additional support and information when they visited their GP following the miscarriage.

“The GP for instance…would have been nice …to be like “Hey you know for you guys there’s these support networks around as well”… or something along those lines.”

- Participant 7, 32 years, 1 miscarriage

The few positive experiences with healthcare providers mentioned by men generally occurred during interactions with nurses or midwives, who tended to take extra steps to ensure a supportive space for both men and women, and ensured both parties understood the procedures or that questions were well answered. Aspects of positive interactions with healthcare services included having continual care and rapport with health care professionals, particularly with specialists and at IVF or early pregnancy specialist clinics.

“The doctor’s clinic, they have all been on our journey and know what we have been through, so they were all very, very supportive. I think they gave us a hug.”

- Participant 2, 37 years, 1 miscarriage

“One nurse …She pulled us aside saying, “you know what, we’re sorry. There’s nothing we can do…If you’re losing your baby, you’re going to lose your baby.” and It was…callous but at least it was an explanation.”

- Participant 10, 29 years, 1 miscarriage

### Importance of sharing their experience

For many men, the opportunity to share their experience of miscarriage, particularly when they had little other support around them, was often cathartic and helpful. Men often found talking, either with family and friends, through support networks where they could share or read or hear others’ experiences, or talking to professionals or a stranger i.e. a counsellor or the interviewer, helped them in coping with their loss and grief. Telling others also gave people the opportunity to provide the help and support men needed.

While none of the men stated explicitly that they suffered anxiety or depression during or after the experience, a number of men spoke of the potential for depression and the need for psychological support for men.

“Somewhere to just get your stuff out there …so you don’t bottle it up so then you don’t get stressed or depressed.”

- Participant 7, 32 years, 1 miscarriage

“You can get really dark in those times…I never ever contemplated suicide…I’d be linking the Lifelines and the Beyond Blues and…those types of things. Which once again I did not seek out but I can see their value.”

- Participant 10, 29 years, 1 miscarriage

#### Family and friends

Experiences and support received from family and friends was mixed across the study. Although men generally felt their loved ones wanted to be supportive and did care, often they focused too heavily on their partner’s wellbeing or did not ask how the men were coping at all.

“It’s also cultural isn’t it…amongst our friends and family …I mean everyone. Most people are concerned about [my partner].”

- Participant 6, 49 years, 2 miscarriages

“…occasionally someone might ask something about it, like even now but that doesn’t really go into conversation. It’s just a question and a simple answer.”

- Participant 7, 32 years, 1 miscarriage

Despite acknowledging friends as trying to be supportive and helpful, several men described friends as not understanding.

“My best mate said it straight up “look talk to me as much as you want but I won’t understand” …If I was ready to talk, he was ready to listen.”

- Participant 2, 37 years, 1 miscarriage

While parents tended to provide more support, they often focused too heavily on the future and encouraging their sons to move forward rather than discussing men’s present grief or the miscarriage.

“…family members saying things like “yeah it’s time for you to move on. You need to get over it now.””

- Participant 1, 31 years, 1 miscarriage

Several men described experiencing their miscarriage whilst living abroad or interstate and that their family were too far away and too removed from the situation to understand or offer support.

“My immediate family is overseas… they knew what’s going on and I think they… I didn’t feel they fully connect to … the emotional rollercoaster we were going through.”

- Participant 9, 43 years, 4 miscarriages

The support men most wanted was within their social networks, and more broadly, was to be shown some acknowledgement, understanding and sensitivity around their loss. They felt sharing their experience, loss and fear with family and friends helped them to grieve. Having family and friends listen and acknowledge the loss or share their own miscarriage story was helpful, while offering their opinion on moving forward, especially when they had no experience of miscarriage, was not always helpful.

“I think friends and family were amazingly understanding…but that relies on being told…talking openly about it helped…Acknowledge that it happened.”

- Participant 10, 29 years, 1 miscarriage

“You get lots of people that say the wrong things to you too…unless they’ve been through exactly what you’ve been through…you don’t really want to hear it (their opinion).”

- Participant 8, 32 years, 1 miscarriage

Several men spoke of their surprise upon sharing their stories and hearing friends and family members had also experienced miscarriage, and hearing how common it was. Men felt discussion of miscarriage to be generally taboo and that this should change within society.

“The first time I heard how many people (are) going through this …But nobody ever talks about it… it would be lovely to be a bit more open …lose the taboo about it… share their experiences.”

- Participant 9, 43 years, 4 miscarriages

“It’s much more common than I think… we believe …people don’t talk about it.”

- Participant 6, 49 years, 2 miscarriages

#### Online support networks and professionals

Men described feeling their own social networks and health care professionals did not understand the experience or were generally focused only on their partner, this was also expected of the man. This led men to independently or with their partner seek out additional external supports online, or confidentially and privately through pregnancy loss organisations and professionals.

“…people may not always know how to support you in these situations…so it would be nice if somebody a bit more professional…to guide you through that…just alleviate the emotional load.”

- Participant 9, 43 years, 4 miscarriages

Men felt additional professional support, including psychologists, telephone counsellors, pastors and life coaches was often needed due to a lack of hospital follow up and no formal supports offered or referred to them individually. Several men described using professional services during this time, although they were generally to address other mental health issues and/or were with their partner. One man was previously diagnosed with anxiety, and two men saw professionals with their wives who suffered mental health illnesses prior to the miscarriage.

“I had a conversation with the local pastor… someone who was sort of disconnected from it all. So he was available to talk about it, so I imagine to someone who isn’t religious …would respond similarly to a counsellor or some sort of psychiatrist.”

- Participant 10, 29 years, 1 miscarriage

“We had a couple of sessions together with that counsellor…. I did find them helpful… just to help look out… generally look out for her essentially.”

- Participant 3, 41 years, 3 miscarriages

More commonly, men found and connected with organisations or support networks through Google or social media. Facebook was a medium which many men felt allowed them to connect and share with others who had experienced miscarriage, and gain support and knowledge. ‘Bears of Hope’, a pregnancy loss support group used to recruit most participants, and ‘Beards of Hope’ which is focused on men, were both mentioned as being beneficial.

“I put up a Facebook post. I can’t remember exactly what I said, but I had friends from high school contacting me. No men, other women. That this happened to me.”

- Participant 10, 29 years, 1 miscarriage

“My wife… got me on to the men’s group …’Beards of Hope’…What the Beards of Hope had, particularly on the Facebook page, was guys just writing up about their experiences. And other people going on and commenting.”

- Participant 2, 37 years, 1 miscarriage

#### Recommendations for resources

In general, men felt there was a strong need for additional male oriented support and resources as most currently available resources were not directed at men.

“I didn’t find a great deal about the father’s side of things.”

- Participant 5, 39 years, 2 miscarriages

Men expressed a need for male orientated information, support networks and professionals with whom to talk. In particular, men described wanting statistics or clear facts or videos from reputable websites detailing causes and complications of miscarriage.

“I did have a thirst for knowledge about it for a while…I found [helpful]. …You know, 1 in 4 people go through this…throwing statistics out there really focused me…umm I really responded to the … hard and fast and short answers.”

- Participant 10, 29 years, 1 miscarriage

Most men felt they would have benefitted from information on ways to support themselves and their partner. They often searched for this information on forums and Facebook sites, reading opinions and stories from others who had experienced miscarriage.

“Obviously anything that helps …dads’ grieving would be good. But I guess just ways or strategies to try and help your wife get through it …would be really good.”

- Participant 5, 39 years, 2 miscarriages

“Guys coming out and asking “My wife’s going through this, how do I look after her?”… a lot of the guys get on and say “you know you need to look after yourself. If you don’t look after yourself there’s not going to be anybody there to look after your wife…if you see this, this and this in yourself you need help.””

- Participant 2, 37 years, 1 miscarriage

Men showed a clear preference for online forums or support sites, or telephone helplines or counsellors.

“Because there’s nothing out there for guys…as in there’s no support groups or let’s have a chat about it or things like that.”

- Participant 10, 29 years, 1 miscarriage

“I think a good idea for them to get a support network out…primarily focused on the males… if there is (one) I didn’t find it.”

- Participant 7, 32 years, 1 miscarriage

Some men detailed the benefit and preference for counsellors or support available by phone or Skype. Attending in person was seen by some as a barrier to receiving this support.

“We did try one [support group]. J and I together …but we actually felt that we were wasting their time because I was the only bloke there.”

- Participant 2, 37 years, 1 miscarriage

During the miscarriage and when their partner was in hospital, men described using their mobile phones to search for information on symptoms and medical procedures. Following the miscarriage, most men felt mobile phones allowed easy access to information and support, whilst maintaining privacy from others, including their partner. They felt additional information and support services should be accessible on mobile devices.

“The first thing you do while you’re at the hospital… you’re on your phone and your googling…all the terms… you know bleeding and stuff like that.”

- Participant 8, 32 years, 1 miscarriage

“I’d probably end up using it predominantly on my phone… I feel that reading this kind of information is very private …I don’t want my wife to kind of react to something I’m looking at…Or possibly bring something up with her.”

- Participant 1, 31 years, 1 miscarriage

All men were grateful to participate in this study and could see the potential to help other men and the need for additional resources for men. All men felt a website providing miscarriage information, resources, support information and links to support services would be very beneficial, provided they linked in with already existing services and ensured information and support was easily accessible and in one communal website.

“…there are so many groups if you all work together to support people you’d have the funds, you’d have the resources that you’d have all that information in one central location…cause now if someone goes and Googles this, they’re having to go through bits and pieces throughout the internet.”

- Participant 4, 44 years, 2 miscarriages

## Discussion

To address the gap in Australian literature, this study aimed to explore the male partner’s experience of miscarriage and their need for additional support at this time. It was not uncommon for male partners to experience feelings of grief of a similar intensity to women. However, whereas women often consider their partner as their central support figure at the time of miscarriage [34], men in this study, commonly reported feeling they could not talk to their partner about their feelings for fear of burdening them further. Expression of grief and loss was also affected by a perceived need for them to be stoic in their support of their partner. This differs to women’s experiences, where they do not report feeling that they have to be strong for their partner. Like women, men commonly reported a lack of emotional support from healthcare and social networks at the time of miscarriage. It appears however that men may have even less support around them at the time of miscarriage, with many stating that healthcare providers, and family and friends directed their acknowledgement and support toward their partners rather than themselves at the time of their loss. Support services and information were also largely targeted at women, leaving men feeling very isolated and alone at the time of miscarriage, and consequently turning to online forums for support and to share their experiences of miscarriage.

The emotional responses and coping mechanisms reported by men in this study generally reflect the findings of the limited international literature. Prioritizing their partner’s wellbeing and grief over their own and being available to support and protect their partner is not uncommon [10, 25–27]. Similar to previous studies, men tended to try to ignore their grief, express it less publically, employ distractions and focus on moving forward more so than their female partners [1,17]. These findings are not surprising given societal expectations around masculinity and the expectation for men to be the pillar of support during times of crisis. This, as seen in this study, often results in men hiding their emotions from their partner and having to deal with their grief alone, findings which have been previously reported in other studies [21, 25, 27, 35]. Men in this study however did not speak of using alcohol, smoking or drugs and medication to distract themselves or cope with their grief, differing to previous study findings [1,17].

These expectations around men’s emotional response to miscarriage grief are only reinforced by the lack of support available to men both within the healthcare system and more broadly, with support predominantly directed at women. The importance to men of being able to share their experience, and help other men share theirs, was clearly evident. In acknowledging men’s role and grief, and discussing miscarriage more widely within society there is opportunity to encourage and enable men to open up and share their experiences and seek appropriate support where needed–if and where that support is available.

In addition, it is important to recognise that men affected by miscarriage also often experienced a loss of identity as a father. Previous international studies have also reported that pregnancy loss signified to many men a loss of opportunity and potentially an inability to have children in the future [25]. This anxiety and concern around future pregnancies and children illustrates the long-term psychosocial impact miscarriages can have on men, and the benefit of providing immediate and ongoing information and support around fertility, subsequent pregnancies, and alternative parenting pathways.

A significant pathway through which men can receive improved acknowledgement and support is their interactions with healthcare professionals. Unfortunately, in line with previous studies with men [10, 21, 22, 25] and women [11, 19, 20], men generally described negative health experiences and interactions with doctors. They reported that their grief and role as a father was unacknowledged, they were ill-informed at the time of the miscarriage and that there was little or no follow up or professional support pathways. Positive interactions largely centered around communications with nurses rather than doctors or at specialist clinics rather than in the hospital emergency department or as an inpatient. Similar findings have been described in previous literature [20, 21]. These services may respond poorly to men due to a cycle of health professionals and programs having adopted traditional social attitudes of pregnancy loss (and pregnancy in general) being a women’s issue and ideas of masculinity which perceive men as not needing support. Further, men do not tend to voice their distress or seek help from health professionals, particularly when support is largely targeted toward women, further reinforcing these stereotypes and attitudes [23, 24]. Further research is required to inform evidence based national guidelines around improved bereavement care in hospitals and by health care professionals in general. Through discussion with men and identification and adoption of services which provide information and support for men, that is both seen as relevant, needed and male-oriented more men will seek these services, and in return more services will be created [23, 24].

Overall, men felt a strong need for additional support resources for men, clearly expressing their preference for online services which were male oriented and mobile device friendly. Websites, online communities or apps allow easy and private use from mobile devices to anonymously share experiences and access information. They also provide the ability to connect with health professionals or other men through phone hotlines, social media, web forums or organised meet-ups. Most men suggested a website, tailored to men’s needs, would be one mechanism that could adequately provide information and support during this time. While some men spoke of their use of, and benefits of counselling, having to attend in person was seen as a barrier. Given the significant psychosocial impact of miscarriage on men, further exploration into the need for, use of and preferences for male-oriented counselling at the time of miscarriage would be useful.

### Strengths and limitations

The major strength of this study is that is one of only four Australian studies to specifically explore men’s experiences of miscarriage and to investigate their support needs [10, 25, 26, 28], and only one of two Australian studies conducted in the last decade [28]. This is also the first Australian study we are aware of to specifically examine men’s health seeking behaviours, support needs and online support seeking behaviours at the time of and following miscarriage.

Almost all men in this study were recruited through a pregnancy loss support group and it would be beneficial for future studies to explore the experiences of men recruited from a broader range of networks and geographical locations. As most men were recruited through this avenue, the sample comprised men already connected with some support services. Within the sample almost all men were born in Australia, limiting the cultural diversity of participants. It is likely that participants with differing cultural backgrounds, whose first language may not be English, and who may or may not have strong family support systems around them, will have differing experiences and support needs. Further research is also required to explore the needs of non-male partners of women affected by miscarriage.

While men in this study did not personally speak of experiencing depression and anxiety as a result of miscarriage, they did understand how this could be a consequence for other men. It is likely, given how common it is for women to experience pathological grief and clinical levels of anxiety and depression in the weeks, months or even years following miscarriage [1, 14], that men may also affected in this way. It should also be acknowledged, given the societal and personal expectations to be strong described by most men in this study, that men may not have felt comfortable to discuss more serious mental health issues with the young interviewer. This may have limited the openness of their responses when sharing their feelings, symptoms or diagnoses of depression or anxiety with the interviewer or other health professionals in the past.

The conduct of interviews by a final year postgraduate medical student is a strength of the study, as it enabled participants to feel confident their interviewer had knowledge of the clinical processes related to miscarriage, yet distinguished her from fully trained clinicians who were often described negatively by the participants. Men described feeling comfortable during the interviews and were grateful to have had the chance to share their experience and were appreciative of the research focusing in this area. However, the interviewer’s female gender and significantly younger age in comparison to most participants may have also had an impact on their responses in terms of disclosure, and the interpretation of data.

### Future implications

Men commonly experience grief and loss around miscarriage of a similar intensity to women, and yet are often unable or disinclined to share their pain with others around them, despite their need for emotional support. It is imperative that men’s loss -as a father and a parent- is also recognised and acknowledged by healthcare providers and within men’s social networks. Currently, there are few guidelines in Australia around provision of bereavement care for parents affected by pregnancy loss. It is important that any bereavement care advice, training or resources aimed at healthcare professionals consider and include the needs of men. Much of the support men need is similar to that highlighted by women in the literature: sensitivity, acknowledgement of their loss, provision of tailored information and follow up care, often focusing on psychological support [11, 19, 20].

Broader scale research is now required to further explore men’s specific support needs and the most acceptable and feasible way of offering tailored support to men. While psychosocial support for miscarriage is limited, most support currently available is tailored and directed to women. It is likely that the types of support, format and medium through which men need support will differ to the needs of women. In this study, due to limited support, men often turned to online forums to give them an outlet to share their experience and connect with other men with similar experiences. Men had a strong preference for easily accessible online or telephone support with professionals who understand men’s experiences and information around caring for their partner, what to expect in future pregnancies and navigating additional parenting and fertility options. This is novel information in Australia and should aid in the development of a range of accessible, tailored supports for men.

Healthcare providers, researchers and society need to facilitate and encourage men to take an active part in pregnancy, and when miscarriage occurs to ensure there is inclusive, and tailored support and information available to men. Failure to include men in the discussion surrounding miscarriage, and pregnancy in general, will continue to perpetuate expectations of masculinity which ignore men as fathers, and do not acknowledge their grief and need for support. Ending the silence and stigma around miscarriage and improving support will undoubtedly be beneficial for both partners.

## Supporting information

S1 Appendix Interview schedule. (PDF)
