# Supplementary material for: ‘There was just no-one there to acknowledge that it happened to me as well’: A qualitative study of male partner’s experience of miscarriage
Source: PLoS One. 2019 May 28;14(5):e0217395. doi: 10.1371/journal.pone.0217395 (PMC6538154; doi:10.1371/journal.pone.0217395)
Supplement: S1 Appendix — (PDF) [file pone.0217395.s001.pdf]

THE UNIVERSITY OF MELBOURNE

The experience of miscarriage from a male partner perspective

QUESTIONNAIRE & INTERVIEW SCHEDULE

Study ID: _______ Date of interview: __________

Recruitment site: ☐ Email ☐ Snowball sampling ☐ Social Media ☐ Support organisation

Interview site: ☐ Phone ☐ Department of General Practice

DEMOGRAPHICS

1. Age: __________

2. Country of birth:

☐ Australia
☐ Other (please specify) _________________

4. What is the highest level of formal education you have completed?

☐ Secondary school
☐ TAFE diploma/certificate
☐ Undergraduate university degree
☐ Post graduate university degree
☐ Other (please specify) ____________

5. What is your current employment status? [tick more than one option if applicable]

☐ Employed full time
☐ Employed part-time
☐ Employed casual
☐ Unemployed (looking for work)
☐ Not in the workforce (not looking for work – i.e. retired, stay at home mother)
☐ Student
☐ Other (please specify i.e. self-employed)..

6. What is your current relationship status?

☐ Single
☐ Married and living with partner
☐ Not married and living with partner
☐ Divorced/separated/widowed
☐ In a relationship but not living with partner

7. How many miscarriages have you and your partner experienced? …………

Interview Schedule

This interview schedule is a guide only. These areas of interest may be revised as interviewing progresses and reordered to optimise the flow of the interview. New topics/areas of interest that stem from earlier interviews may be added to the schedule.

***Preamble**:*
We know miscarriages can be a very distressing experience and our purpose is not to cause you any further distress. There is very little research that has focused on male partner's experience of miscarriage and how miscarriage affects them. The focus of this study is on your experiences with your family and friends and healthcare professionals at the time, and how those experiences or interactions shaped your experience of miscarriage. We want to use this information, along with your recommendations, to look at ways we can better support men who are affected by miscarriage.

1. Tell me about your experience of miscarriage
[Prompts]
- Your story
- Feelings
- Role was at the time? i.e. to support my partner? to stay strong for my partner?
- What effect did the miscarriage have on your relationship with your partner?

2. Support and acknowledgement
- Did you feel there was acknowledgement of *your* loss?

3. Tell me about the social support you received at this time
- Who
- Kinds of support
- Helpful
- Unhelpful
- Did this experience of miscarriage differ from other experiences of miscarriage you had had prior to this, in terms of healthcare provider and social support?

4. Tell me about your healthcare experience at this time
- Who you saw
- Information they gave
- Advice
- Follow-up – any?
- Referrals to counsellor?

5. Tell me about the supports and resources that were offered to you at this time
- Any other information you would have liked
- Recommendations for healthcare providers
- Recommendations for friends and family

6. Positive experiences
- One or more positive experiences
- In healthcare
- With friends and family

THE UNIVERSITY OF MELBOURNE

Website

We are thinking of building a website to provide pregnancy loss information and advice. In order to build this website we need to know what information and advice would be useful for users.

Internet Search Behaviours

7. Did you do an Internet searching when you and your partner experienced a miscarriage/s?

8. What search terms did you use?

9. What sites did you go to?

10. What was your experience with these sites? Did you find the information you were looking for?

11. What information was missing? How would it have helped?

12. Did you download any information, brochures etc.? Why/Why not?

Explain what we would like to provide in a website i.e. a pregnancy and pregnancy loss information and advice website which also houses a research and funding platform. Show the participants the Tommy's website and the type of information it provides.

Our website

13. What sort of information would you like to see on this website?

14. What sort of other information would you like linked to this website?

15. What sort of tone would you like the website to use i.e. formal, relaxed, clinical, easy to understand etc.?

16. What device would you use to access a website like the one we are talking about? *(e.g. mobile, home computer, tablet etc.)*

17. Any further comments?

18. Housekeeping
    - Contact details: confirm if permission given for further contact
    - How did they feel about the interview?
